# Obesity and Dose of Anti-cancer Therapy: Are We Sure to Be on the Right Track in the Precision Medicine Era?

**DOI:** 10.3389/fmed.2021.725346

**Published:** 2021-09-24

**Authors:** Tania Rossi, Erika Bandini, William Balzi, Francesco Fabbri, Ilaria Massa, Roberta Maltoni

**Affiliations:** ^1^Biosciences Laboratory, IRCCS Istituto Romagnolo per lo Studio dei Tumori (IRST) “Dino Amadori”, Meldola, Italy; ^2^Healthcare Administration, IRCCS Istituto Romagnolo per lo Studio dei Tumori (IRST) “Dino Amadori”, Meldola, Italy

**Keywords:** obesity, body surface area, body mass index, treatment, cancer therapy, cancer

## Introduction

Obesity, defined in adults by a body mass index (BMI) greater than or equal to 30 kg/m^2^ is a growing public health issue, affecting mainly Western countries. In the past, adipose tissue was considered as an inert component with a mere lipid storage function but to date it is recognized as a real organ with metabolic functions. Its relation with increased cancer risk and influence on anti-cancer therapy is not new, especially in association with the low-grade inflammation that characterizes adiposity (1–3). Therefore, adipose tissue cannot be ignored when anti-cancer therapy dosage is calculated. The only current method for cancer therapy dose calculation considers body surface area (BSA), which is not a surrogate of obesity and doesn't take into account any inter-individual variable, resulting in floating effects. Integration of BMI has demonstrated to reduce chemotherapy-induced toxicity, but the formula remains not exhaustive (4).

Here, we criticize the use of BSA and BMI for cancer-therapy dose adjustment, highlighting the need to develop a comprehensive algorithm that could dramatically improve the personalized medicine concept in oncology.

## Shortcomings of BSA-Based Anti-Cancer Therapy Dose Calculation

The BSA formula was introduced in the 1950s for drug dose adjustments, based on the assumption that pharmacological processes are related to body size. Despite several BSA formulas having been proposed over time (5–9), none of these takes into account obesity, remaining a bi-dimensional estimation whose shortcomings are known from at least 25 years (10, 11).

Bins et al. (12) criticized BSA-based chemotherapy dose adjustment, depicting it as a very precise estimation but with no accuracy, hence without medical value. Indeed, BSA is not a measure, but rather an estimation among the most difficult anthropometric procedures (13).

More recently, critiques concerning the negligence of sexual differences during dose calculations have been leveled by the European Society of Medical Oncology (ESMO) (14). This shortcoming is flagrant, since biological sexual disparities have been deeply investigated and are known to influence cancer development and treatment, leading to a proper “sexual dimorphism in cancer” (15). Data from different cancer types have clearly demonstrated that the female population is more susceptible to chemotherapy-derived toxicity. This disparity is the consequence of differences in drug clearance between sexes, and the higher percentage in men of metabolically active fat-free body mass (FFM) overall compared to women (16–20). In addition, men and women differ in drug absorption and distribution (21). Therefore, in addition to neglecting obesity, BSA does not take into account interpersonal variability and is considered an outdated formula that should be reconsidered (12). Alternative dosing strategies have been hypothesized, but their utilization in the common medical practice have been considered not practicable due to the limited types of cancer and settings tested.

To take into consideration obesity in anti-cancer therapy dose, several corrections to the BSA formula have been proposed for dose adjustments in obese patients (4). For instance, in the early 2000s, Portugal demonstrated that correction of BSA with BMI, which is used as a surrogate for body fat, could be helpful to overcome BSA limitations, significantly reducing the chemotherapy-induced toxicity (22). However, like BSA, BMI is calculated by taking in consideration only height and weight, hence still bypassing inter-patient variability. One major flaw of BMI and BSA is the failure to account for body composition. This has been defined as the proportions and distribution of lean and fat tissues in the human body, and it is becoming an emergent aspect in oncology (23). For instance, for what concerns BMI, a bodybuilder with a high percentage of muscle tissue and low percentage of adipose tissue could have the same BMI as obese patients (24). Moreover, body composition could be influenced by severe depletion of the muscle tissue in obese patients, commonly known as sarcopenia. It has been shown that, on average, 25% of obese patients diagnosed with solid tumors present sarcopenic obesity associated with higher mortality and higher complications following cancer therapy and surgery (23). The high toxicity may be due to the BSA-based chemotherapy dose adjustment, since large BSA typical of obese patients corresponds to high drug dose which is disproportionate for a body with very depleted lean mass (25). Further evaluations through diagnostic imaging techniques are considered the only valid measurements to gain precious information concerning the body composition.

Another complex variable is related to the cancer-affected organ and its anatomy. For instance, a recent meta-analysis by Petrelli et al. (26) grouping more than 6 million patients from 203 studies, found that obesity, intended as BMI ≥ 30, was associated with reduced overall survival (OS) and cancer-specific survival (CSS) as well as increased risk of recurrence. Strikingly, obese patients diagnosed with lung cancer, renal cell carcinoma and melanoma showed improved survival compared to non-obese patients affected by the same cancer type. This phenomenon, known as “paradox obesity,” is not yet fully elucidated. Explanations are still controversial due to the complexity of the networks involved in the adipose tissue biology, going beyond BMI formula. In the same manner, it has been hypothesized that in obese renal cell carcinoma patient the white perinephric adipose tissue could act as a reservoir of immune cell (TH1 cells, Tregs, dendritic cells, and type 1 macrophages) (27, 28). Paradox obesity was evident in HER2-positive breast cancer (BC) patients based on the stage, since higher BMI was associated with reduced OS and disease-free survival (DFS) in the early setting, but improved OS and progression-free survival (PFS) in advanced stage BC (29). These evidences highlight a need to better understand the biological basis of obesity in different settings and tumor subtypes.

Obesity proved to play a crucial role in incidence and mortality of BC, which is considered as one of the most commonly diagnosed tumors in the female population with over 2 million new cases in 2018. Studies in literature demonstrate that obesity in BC patients is associated with increased tumor dimension, lymph node positivity, metastasis development and shorter OS and DFS, as well as resistance to therapies (30, 31). However, in early-stage BC patients with aggressive biological subtype treated with adjuvant chemotherapy the impact of higher BMI had no influence on prognosis (32), suggesting the need to better understand the role of obesity based on pathological and biological features of BC.

Interestingly, obesity-related proteins have been investigated by Diao et al. (33) through the development of an obesity-related protein score (ORPS), in order to identify helpful markers to predict BC risk. In particular, resistin (RETN) and C-reactive protein (CRP) were found upregulated in pre- and post-menopausal women, while soluble leptin receptor (sOB-R) and adiponectin (ADP) were observed downregulated compared to healthy subjects. In premenopausal women, insulin-like growth factor binding protein-3 (IGFBP-3) was reported downregulated compared to healthy volunteers.

The aggressiveness of BC in obese patients could be imputable to the complex biologic interaction between the primary tumor and the adipokines produced by the adipose tissue (34), among which leptin, as reported by clinical and experimental findings. For instance, leptin, together with interleukin-6 (IL-6) and tumor necrosis factor α (TNFα), has been demonstrated to decrease the activity of tamoxifen metabolites in obese patients (35) supporting the role of adipocyte-derived secretome and fat tissue in this disease.

The adjustment of chemotherapy dose using BSA has been heavily criticized in BC due to the neglecting of fat distribution, whose localization (visceral, subcutaneous, intern) is highly variable between individuals. In this context, Iwase et al. (36) demonstrated that BC patients with higher visceral fat had shorter DFS after neoadjuvant chemotherapy, especially in post-menopausal women.

A study by Pfeiler et al. (37) showed that obese BC patients treated with anastrozole had a disease recurrence risk increase of 60% other than a doubled risk of death, compared to those with normal weight. Indeed, dose adjustment is not performed for drugs among which hormonal therapies (tamoxifen, aromatase inhibitors (AI), fulvestrant), cyclin dependent kinase (CDK) 4/6 inhibitors, methotrexate, cyclophosphamide and monoclonal antibodies, leading to possible underdosing or toxicity in obese and underweight BC patients, respectively. Fixed-dose criticisms are known from the early 2000s, when Plumridge and Sewell (38) proposed dose-banding to overcome this issue. Taken together, these findings support the need to reconsider the validity of fixed-dose.

## Conclusions

In the precision medicine era, physicians spend great effort to address the treatment strategy based on tumor clinical-pathological, biological and molecular features. However, although the shortcomings of the BSA-dose adjusting method have been known for decades, up to now this criticism is still ongoing.

Herein, we highlight that BSA, which is currently and widely used, is not sufficient for dose calculation in cancer patients, and correction of this formula with BMI has limited value. Conversely, a new algorithm should be developed, taking into account, besides height and weight, inter-patient variable parameters such as sex, age, body composition, setting, type of cancer and its clinical-pathological, biological and molecular features, in order to improve the efficiency of anti-cancer treatment in the precision medicine epoch. In addition, a deeper understanding of the biological processes involving the adipose tissue would be helpful to sharpen this formula.

## Author Contributions

TR and RM conceived the idea. TR, EB, FF, WB, IM, and RM contributing to write the first draft. TR, EB, FF, and RM revised the manuscript. All authors contributed to the article and approved the submitted version.

## Conflict of Interest

The authors declare that the research was conducted in the absence of any commercial or financial relationships that could be construed as a potential conflict of interest.

## Publisher's Note

All claims expressed in this article are solely those of the authors and do not necessarily represent those of their affiliated organizations, or those of the publisher, the editors and the reviewers. Any product that may be evaluated in this article, or claim that may be made by its manufacturer, is not guaranteed or endorsed by the publisher.
